# Spinocerebellar ataxia Type 7: clinical and genetic study of a new Moroccan family (case report)

**DOI:** 10.11604/pamj.2021.38.162.27262

**Published:** 2021-02-12

**Authors:** Fatima Zahra Bouzid, Maria Mansouri, Chaikhy Abdelaziz, Nisrine Louhab, Sablonniere Bernard, Isabelle Strubi-Vuillaume, Kenza Dafir, Nisrine Aboussair

**Affiliations:** 1 Genetics Department, Clinical Research Center, University Hospital Centre Mohammed VI, Marrakech, Morocco,; 2 School of Medicine and Pharmacy of Marrakech, Cadi Ayyad University, Marrakech, Morocco,; 3 Private Ophthalmology Practice Agadir, Agadir, Maroc,; 4 Neurology Department, University Hospital Centre Mohammed VI, Marrakech, Morocco,; 5 Biochemistry and Molecular Biology Pole, Department of Neurobiology Biology Pathology Center, Lille University Hospital, Lille, French

**Keywords:** Case report, spinocerebellar ataxia type 7, ATXN7 gene, CAG repeats

## Abstract

Spinocerebellar ataxia type 7 (SCA7) is a rare autosomal dominant neurodegenerative disease. Its clinical presentation is a progressive cerebellar ataxia associated with cone and retinal dystrophy. The CAG repeat expansion in the ataxin-7 gene (ATXN7) causes spinocerebellar ataxia type 7 - a mutation that results in the degeneration of the brain stem cells, retina and cerebellum. We report in this study the clinical and genetic features of a new Moroccan family of SCA7, from the South of Morocco. We performed the molecular genetic testing to confirm the diagnosis of SCA7. The objective of this study is to report a new Moroccan case of SCA7 and to illustrate the role of the geneticist in the diagnosis, management and development of genetic counseling of SCA7 disease.

## Introduction

Spinocerebellar ataxia type 7 (SCA 7) is a rare neurodegenerative disease (100000) with a progressive cerebellar ataxia, ophthalmoplegia and retinal degeneration. Its other clinical signs may be less common, such as sensory abnormalities, dysarthria and dysphagia [1]. The disease-causing mechanism is the expansion of the polyQ tract of the ATXN7 gene, which codes for a predominantly nuclear protein ataxin 7 that shuttles between the nucleus and cytoplasm. A normal number of CAG repeats is between 7 and 27. However, pathogenic alleles contain 37 to 460 repeats [2]. A study has reported the presence of phenotype-genotype correlation in SCA7 [3], a repetition greater than 37 CAG repeats is correlated with disease severity and inversely correlated to the age at the onset. Therefore, the expansion size can be a marker of severity.

The SCA7 is characterized by intra-clinical variability. The onset of clinical symptomatology ranges from infantile - considered as the severe form with an early death to late onset in the elderly of an isolated and slowly progressive ataxia. Thus, the clinical course and the date of onset of symptoms are closely related to the number of CAG repetitions in the SCA7 gene [4]. We report a new Moroccan patient with SCA7, who was referred on November 4^th^, 2016 to our genetics department in the clinical research center of Mohammed VI University Hospital of Marrakech. After neurological and ophthalmological examinations of all family members, we proceeded to a genetic consultation in our department and we established the adequate family pedigree, which illustrated an autosomal dominant inheritance pattern. In order to illustrate the role of the geneticist in the diagnosis, management and development of the genetic counseling of the SCA7, we reported a new Moroccan case of SCA7.

## Patient and observation

**Figure 1:**
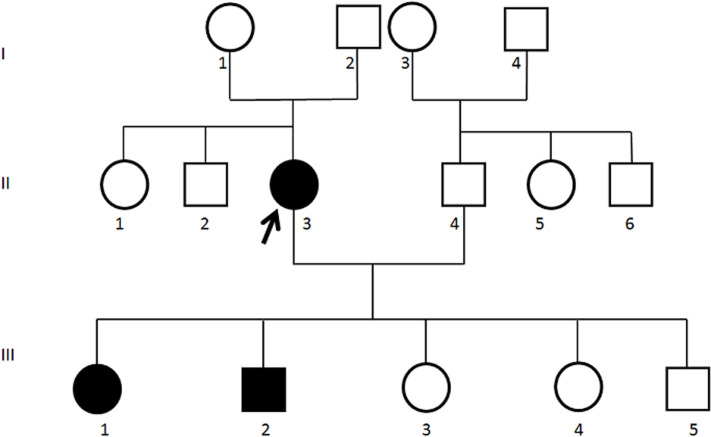
family pedigree showed an autosomal dominant inheritance pattern

A 40-year-old married woman (the probands: II3), non-consanguineous, mother of five children. Her family history was remarkable; two of her children were diagnosed with spinocerebellar ataxia (Figure 1). In 2011, the patient developed a progressive cerebellar ataxia associated with coordination disorder and a significant decrease in visual acuity. In the clinical findings, the patient presented an ataxia, impaired coordination with vivid and diffuse osteotendinous reflexes, a negative Babinski sign and a positive Hoffman´s sign. In contrast, muscle tone, deep sensitivity and thermo-pain were normal. The patient did not present hollow feet or scoliosis. The ophthalmologic examination revealed a decrease in visual acuity with significant macular damage and a bilateral optic atrophy. The routine laboratory testing and brain computed tomography (CT) and magnetic resonance imaging (MRI) detected isolated pontocerebellar atrophy.

The daughter III1 was symptomatic up to 19 years old, with a progressive ataxia and an ophthalmoplegia. The neurological examination revealed a vivid and diffuse osteotendinous reflexes. Also, the son III2 was symptomatic up to 17 years old with a progressive ataxia, ophtalmoparesia and coordination disorder. The clinical data including spinocerebellar ataxia, maculopathy, optic atrophy and the autosomal dominant inheritance manner have supported the clinical diagnosis of SCA7.

**Figure 2:**
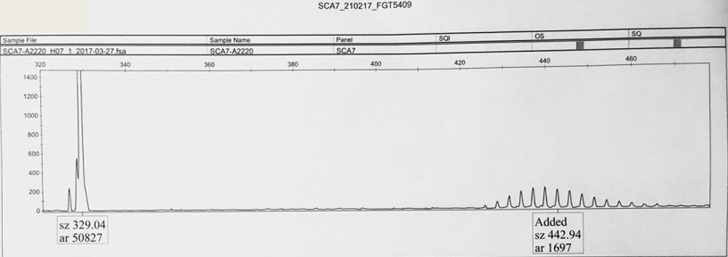
electrophoretic profile of PCR-amplified CAG repeat expansions (image showed our patient´s two SCA7 alleles: the normal allele at 329 bp (10 triplets) and the expanded allele at approximately 443 bp (approximately 50 triplets))

All family members included in this report case signed a written informed consent before DNA extraction. We conducted Genomic DNA purification from peripheral blood samples in our genetic department in the clinical research center, Mohammed VI University Hospital of Marrakech. We amplified the CAG repeat region in the ATXN7 gene using a fluorescent polymerase chain reaction (PCR) amplification with primers surrounding the repeated region of interest followed by capillary electrophoresis. The fragments analysis revealed the presence of a heterozygote allele with pathological expansion of the CAG triplet of the tested locus, which confirmed the diagnosis of spinocerebellar ataxia type 7 in our patient (the probands: II3) (Figure 2).

## Discussion

Spinocerebellar ataxia type 7 (SCA7) is one of several autosomal dominant ataxias characterized by neuronal loss, usually in the cerebellum and brainstem. The cause of SCA7 is a heterozygote mutation of the CAG repeats expansion [5]. Generally, the CAG repeat in SCA7 patients expands from 37 to 460 triplets. We diagnosed the SCA7 in the probands II3 by the detection of a heterozygous abnormal CAG trinucleotide repeat expansion in ATXN7 using molecular genetic testing. We found the mutation of the CAG repeat expansion at heterozygous state in the ATXN7 gene, which confirmed the spinocerebellar ataxia type 7 diagnosis. The family originates from Agadir, Morocco. It is the first Moroccan family with SCA7 in the South of Morocco. There are a few SCA7 reports from Algeria, Angola, Cape Verde, Liberia, Mali, Morocco, South Africa, Tunisia and Zambia [6,7].

The clinical features of the family members included in this report were similar to those reported in previous studies [6,7]. The size of the CAG repeats and the age of onset were inversely correlated. Our patient had her first symptoms at 37 years old and the molecular analysis had shown 50 expansions of CAG repeat, but her children had their symptoms at the age of 11 and 17 years old. Other studies showed that a larger expansion was associated with onset at an earlier age, shorter life expectancy and higher frequency of decreased vision [7]. We deduced a clinical anticipation and complete penetrance of the SCA7 and we expect to have a molecular confirmation for the other patients once they request it. Genetic counseling can inform parents that the disease has a 50% risk of transmitting the pathogenic variant to each child. Indeed, two of the children of the proband, 19 and 17 years old respectively, III1 and III2 were symptomatic and presented the clinical diagnostic criteria of SCA7. Since DNA from the parents of the proband II3 was not available, it was not possible to determine if the mutation was inherited or de novo.

Meanwhile, the management of affected individuals remains supportive, as no known therapy to delay or halt the progression of the disease exists. In our case, all the patients benefited from ophthalmologic, kinesitherapy, and neurological management with psychological support and adequate genetic counseling.

## Conclusion

A few descriptions of the clinical phenotype and molecular genetics of the SCAs are available from the African continent. We presented a new Moroccan family with typical clinical features and a molecular testing that revealed a pathogenic heterozygote CAG repeat and we demonstrated the role of geneticist in the diagnosis, management and development of the genetic counseling of patients with SCA7.
